# The Inhibitory Tendon-Evoked Reflex Is Increased in the Torque-Enhanced State Following Active Lengthening Compared to a Purely Isometric Contraction

**DOI:** 10.3390/brainsci10010013

**Published:** 2019-12-23

**Authors:** Vincenzo S. Contento, Brian H. Dalton, Geoffrey A. Power

**Affiliations:** 1 Department of Human Health and Nutritional Sciences, College of Biological Sciences, University of Guelph, Guelph, ON N1G 2W1, Canada; vcontent@uoguelph.ca; 2 School of Health and Exercise Science, University of British Columbia, Kelowna, BC V1V 1V7, Canada; brian.dalton@ubc.ca

**Keywords:** electromyography, history dependence of force, residual force enhancement, eccentric, golgi tendon organ, afferent

## Abstract

Residual torque enhancement (rTE) is a history-dependent property of muscle, which results in an increase in steady-state isometric torque production following an active lengthening contraction as compared to a purely isometric (ISO) contraction at the same muscle length and level of activation. Once thought to be only an intrinsic property of muscle, recent evidence during voluntary contractions indicates a neuromechanical coupling between motor neuron excitability and the contractile state of the muscle. However, the mechanism by which this occurs has yet to be elucidated. The purpose of this study was to investigate inhibition arising from tendon-mediated feedback (e.g., Golgi tendon organ; GTO) through tendon electrical stimulation (TStim) in the ISO and rTE states during activation-matching and torque-matching tasks. Fourteen male participants (22 ± 2 years) performed 10 activation-matching contractions at 40% of their maximum tibialis anterior electromyography amplitude (5 ISO/5 rTE) and 10 torque-matching contractions at 40% of their maximum dorsiflexion torque (5 ISO/5 rTE). During both tasks, 10 TStim were delivered during the isometric steady state of all contractions, and the resulting tendon-evoked inhibitory reflexes were averaged and analyzed. Reflex amplitude increased by ~23% in the rTE state compared to the ISO state for the activation-matching task, and no differences were detected for the torque-matching task. The current data indicate an important relationship between afferent feedback in the torque-enhanced state and voluntary control of submaximal contractions. The history-dependent properties of muscle is likely to alter motor neuron excitability through modifications in tension- or torque-mediated afferent feedback arising from the tendon.

## 1. Introduction

Residual torque enhancement (rTE) is a history-dependent muscle property that results in an increase in steady-state isometric (ISO) torque production following an active lengthening contraction as compared to a purely isometric contraction at the same muscle length and level of activation [1,2,3]. The presence of rTE has been demonstrated in vitro, from the level of the sarcomere [4] to whole muscle preparations [5], and at the single muscle fiber level [6] to the whole muscle level, via electrical stimulation [7,8] and submaximal and maximal voluntary isometric contractions (MVCs) in humans [2,9,10]. Although numerous reports have focused on the basic mechanical mechanisms of rTE [5,11,12,13,14], the neural consequences of rTE have been largely underappreciated. Recent studies have reported that, during voluntary contractions, rTE may be linked to excitability modifications within the corticospinal pathway [9,15]. For example, rTE led to an increase in corticospinal excitability during plantar flexion MVCs [15] and a decrease in spinal excitability during submaximal steady-state dorsiflexion contractions [15]. However, it is not clear what factors may be modulating these changes in excitability.

Sypkes et al. [15] proposed that reduced spinal excitability during a condition of enhanced muscle force production capacity may be corresponding to greater inhibition of the agonist motor neuron pool arising from tendon-mediated feedback or, more specifically, the Golgi tendon organ (GTO) [15]. The GTOs are located in series with extrafusal muscle fibers at the interface of the musculotendinous junction (MTJ) and act to sense tension produced by its corresponding activated muscle [16,17,18]. Rising muscle tension increases the firing of the Ib afferent neurons that project from the GTO to a variety of targets within the central nervous system [17,19], including inhibitory interneurons synapsing onto the agonist motor neuron pool [20,21]. An effective mode to assess the efficacy of inhibitory pathways onto the motor neuron pool is using tendon electrical stimulation (TStim) to elicit a short latency reflex [22,23]. Applying TStim during times of increased muscle tension (i.e., rTE state) may elicit increased Ib inhibitory reflex parameters [22,24,25]. Recently, using this technique, we have shown that the tendon-evoked inhibitory reflex is reduced in the shortening-induced residual torque depressed state [23]. The opposite may be true for a condition of enhanced torque production (e.g., rTE). For a review on residual torque depression, please see Chen et al. [26].

Residual torque enhancement can be measured as the increase in torque following active lengthening in the isometric steady-state phase compared to purely ISO contractions during activation-matched tasks (i.e., electromyography), and it has been observed during both submaximal and maximal voluntary activation [27,28,29]. It is proposed that rTE occurs due to a stiffening and shortening of titin’s free spring length in the presence of calcium and cross-bridge cycling [27], effectively increasing the contribution of passive force to total force following active lengthening. Therefore, while matching torque, the increase in passive tension associated with rTE [5,30] requires less muscle activation as compared with a purely ISO contraction [28,29]. This activation reduction is regularly measured through a decrease in electromyography (EMG) and has been observed in both submaximally and maximally activated contractions [3,7,28,31]. These two methods of evaluating rTE—activation matching and torque matching—provide a unique approach to investigate the role of tension-mediated afferents on previously observed reductions in spinal excitability.

The purpose of this study was to determine how the tendon-evoked inhibitory reflex is modified between the rTE and ISO states and to discuss its implications in the reduction of motor neuron pool excitability in the rTE state. During activation-matched ISO and rTE contractions, muscle tension in the isometric steady state is expected to be greater, whereas muscle tension is expected to be equivalent during torque-matched ISO and rTE contractions. If previously observed reductions in agonist motor neuron pool excitability during rTE contractions are in fact modulated from tendon-mediated feedback, we hypothesize that increases in TStim-elucidated reflex parameters (such as amplitude) should be apparent in rTE contractions during isometric steady state compared to purely ISO contractions for the activation-matching trials but not the torque-matching ones.

## 2. Materials and Methods

### 2.1. Participants

Fourteen healthy male participants with a mean age of 22 ± 2 years, height of 180 ± 6 cm, and mass of 81.8 ± 13.2 kg with no prior history of neuromuscular disease or ankle joint injuries were recruited from the university population. Participants gave written informed consent prior to testing. All procedures were approved by the Human Research Ethics Board of the University of Guelph (REB: 15NV008).

### 2.2. Experimental Set Up

A HUMAC NORM dynamometer (CSMi Medical Solutions, Stoughton, MA, USA) was used to record torque, angular velocity, and position. Each participant sat with their right hip and knee angles set at 110° and 140° (180° = full extension), respectively. Joint angles were measured using a goniometer. The right knee was immobilized just proximal to the patella with the dynamometer’s leg restraint preventing hip flexion and a cushion positioned beneath the distal hamstrings preventing hip extension, while movement at the torso was restricted with a four-point seatbelt harness. The right foot was fixed to the dorsi/plantar flexor adaptor with one inelastic strap secured over the ankle and another across the mid-distal portion of the metatarsals. The dynamometer’s maximum ankle dorsi- and plantar flexion angles were set to 0° and 40° plantar flexion (PF; 0° = neutral), respectively, allowing for 40° of ankle excursion.

Locations for the surface EMG electrodes (Ag/AgCl, 1.5 × 1 cm: Kendall, Mansfield, MA, USA) were prepared by shaving and cleaning the skin with alcohol swabs. The active electrode was placed over the tibialis anterior (TA) approximately 7 cm inferior and 2 cm lateral to the tibial tuberosity, and a reference electrode was placed inferiorly, adjacent to the active electrode in line with the muscle fibers. To record antagonist activity, the active electrode was placed on the soleus, along the midline of the leg approximately 2 cm inferior to the border of the heads of the gastrocnemii, and a reference electrode was placed inferiorly, bordering the active electrode. A single ground electrode was positioned over the patella.

Surface EMG, torque, angular velocity, dynamometer position, and stimulus trigger data were digitized using a 12-bit analog-to-digital converter (PowerLab System 16/35, ADInstruments, Bella Vista, Australia) and analyzed with Labchart software (Labchart, Pro Modules 2014, version 8). Torque angular velocity and position as well as EMG data were recorded at a sampling rate of 1000 and 2000 Hz, respectively. The EMG data were bandpass filtered using a digital filter (10–1000 Hz).

### 2.3. Peripheral Nerve Stimulation

**Figure 1:**
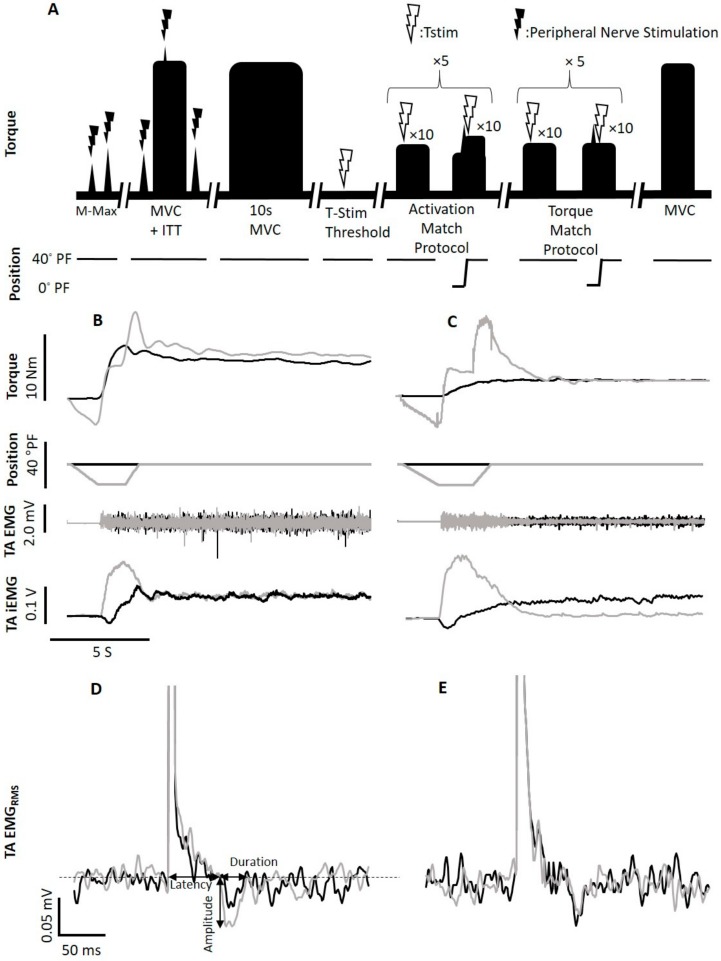
Timeline and raw data traces. Schematic timeline of experimental procedures (**A**). To establish maximum compound muscle action potentials (Mmax) for the tibialis anterior (TA) and soleus, peripheral nerve stimulation was performed on the deep branch of the common fibular and tibial nerves, respectively. A 10 s maximum voluntary contraction (MVC) was followed by an initial MVC performed with the interpolated twitch technique (ITT) to assess maximum torque and voluntary activation. Tendon electrical stimulation (TStim) was applied at increasing currents until perceptual threshold (PT) was found, which was defined as the minimum current intensity that induced a tingling or tapping sensation that was detectable to the participant. Stimulation intensity was then increased to 6 × PT, a current at which participants reported a muscular sensation, including a tugging or pulling at the muscular insertion, a deep tingling near the cathode, or a muscle twitch. If a visible muscle twitch was induced, stimulation intensity was reduced to the maximum current that failed to produce a visible muscle twitch. This stimulation intensity was used for all subsequent trials. Participants then performed 10 pairs of activation-matched dorsiflexion trials (**B**) consisting of an isometric (ISO) trial (black trace) followed by a residual torque-enhanced (rTE) trial (gray trace). These contractions were performed at 40 ± 5% of the participant’s maximum TA activation, and 10 TStims were applied during the isometric steady state of each contraction. Participants then performed 10 pairs of torque-matched dorsiflexion trials (**C**) consisting of an ISO trial (black trace) followed by an rTE trial (gray trace). These contractions were performed at 40% of the participants maximum dorsiflexion torque, and 10 TStims were applied during the isometric steady state of each contraction to evoke an inhibitory reflex (activation-matched trial (**D**), torque-matched trial (**E**)). Lastly, participants were instructed to perform a final MVC to assess for fatigue. A resting period of 5 min was given to participants after all contractions, and each contraction was performed with visual feedback as well as verbal encouragement.

To test the voluntary activation of the dorsiflexors (see next section) and obtain compound muscle action potentials (M-waves) from the TA and soleus, peripheral nerve stimulation was delivered transcutaneously with a standard clinical bar electrode (Empi, St. Paul, MN, USA) coated in conductive gel. The deep fibular nerve, innervating the dorsiflexor muscles, was located by palpating the head of the fibula and moving posteroinferiorly until the nerve was identified. Stimulation distal to the bifurcation of the common fibular nerve was ensured in order to limit activation of the peroneal muscles. The tibial nerve was stimulated via a bar electrode positioned within the popliteal fossa to maximize the M-wave. All stimulations were delivered as a single square-wave pulse from a constant-current, high-voltage stimulator (model DS7AH, Digitimer, Welwyn Garden City, Hertfordshire, UK). Voltage and pulse width were set to a maximum of 400 V and 200 μs, respectively. The current was increased incrementally until a plateau was reached for the peak-to-peak amplitude of the resting M-wave (Mmax). To ensure consistent activation of all motor neurons throughout the experiment, the current was then increased to a supramaximal level, equivalent to 110% of that required to generate Mmax (range: 20–200 mA and 25–250 mA for deep fibular and tibial nerves, respectively). An overview of all experimental procedures is provided in Figure 1A.

### 2.4. Maximum Voluntary Contraction and Voluntary Activation

Voluntary activation of the dorsiflexors was assessed during brief MVCs (~5 s) performed twice, separated by 4 min of rest both prior to and following the experimental trials. The interpolated twitch technique was used to evaluate voluntary activation [32]. The torque resulting from peripheral nerve stimulation delivered during the plateau phase of the MVC was compared to a resting twitch evoked 1–2 s after relaxation. The level of voluntary activation was calculated as follows: voluntary activation (%) = [1 −(interpolated twitch torque/resting twitch torque)] × 100%. Participants were encouraged verbally and provided visual feedback of torque output during all MVC attempts [33]. All participants were required to reach a minimum of 95% voluntary activation in order to be included in the study and were given five minutes of rest following the qualifying MVCs before continuing with the experiment.

### 2.5. Determining Submaximal Muscle Activation

To determine the submaximal integrated EMG (iEMG) and torque targets, participants were instructed to perform a 10 s dorsiflexion MVC at an ankle angle of 40° PF. The average iEMG and torque collected between 6 and 8 s was then used to determine the 40% submaximal iEMG and torque targets [23] (Figure 1B). For activation-matched contractions, a ± 5% window was calculated about the 40% iEMG target, and participants were instructed to maintain their iEMG amplitude within set guidelines marking this target window [23].

### 2.6. Tendon Electrical Stimulation

Percutaneous TStim was used to induce tendon-evoked inhibitory agonist reflexes. This technique involved percutaneous square-wave electrical stimulation of the tendon near the MTJ and evoked a short-latency (<50 ms) reflexive inhibition in the agonist muscle. This reflex has been demonstrated for several upper and lower limb muscles [22,23,24,25] and is thought to be mediated via Ib spinal pathways owing to the short latency and the polarity of the response, which is consistent with Ib autogenic inhibition [22,23]. However, contributions from other sources, such as muscle or tendon type III afferents, cannot be ruled out completely [25]. Still, non-GTO origins of TStim, specifically cutaneous receptors overlying the tendon or muscle stimulation via current spread, were excluded in prior studies [24]. Previous reports [22,34] have demonstrated that indwelling electrical stimulation of the tendon evokes an inhibitory response with similar characteristics as percutaneous stimulation, indicating that the percutaneous tendon-evoked reflex technique used here is most likely tension-mediated via GTO afferents. Ag/AgCl electrodes (1.5 × 1 cm: Kendall, Mansfield, MA, USA) were used for TStim in order to generate tendon-evoked inhibitory reflexes. The cathode was placed near the MTJ of the TA, and the anode was placed over the distal tendon at the level of the malleoli [23]. Single stimuli were presented with a constant-current, high-voltage stimulator (DS7AH). Voltage was set to a maximum of 400 V and pulse width to 200 μs. The stimulation protocol was initiated with the detection of perceptual threshold (PT), which was defined as the minimum current intensity that induced a tingling or tapping sensation that was detectable to the participant (6.63 ± 2.13 mA) [23]. Stimulation intensity was then increased to 6 × PT, a current at which participants have reported a muscular sensation, including a tugging or pulling at the muscular insertion, a deep tingling near the cathode, or a muscle twitch [9,23]. If a visible muscle twitch was induced, stimulation intensity was reduced to the maximum current that failed to produce a visible muscle twitch [23]. This stimulation intensity was used for all subsequent trials (5.2 ± 0.90 × PT).

### 2.7. Experimental Procedures

Each rTE trial was preceded by an ISO trial, and protocol A was followed by protocol B. Five rTE trials and five ISO trials were performed for each of the two protocols for a total of 20 contractions. Participants were provided visual feedback of the iEMG and torque amplitudes on a computer monitor and were verbally encouraged to match the target as closely as possible during all submaximal contractions. Four minutes of rest separated all submaximal contractions.

### 2.8. Protocol “A”: Activation-Matching Condition

For each rTE trial, the protocol consisted of a 40% iEMG contraction involving a 2 s isometric phase at an ankle angle of 90°, a 1 s isokinetic lengthening phase (angular velocity: 40°/s) and ~20 s isometric phase at 40° PF (Figure 1B). A series of 10 TStim pulses were delivered at random 1–4 s intervals [9,23] during the isometric phase at 40° PF. During the ISO trials, an isometric dorsiflexion contraction corresponding to 40% iEMG was performed for ~23 s at an ankle angle of 40° PF, with a similar pattern of stimuli delivered as described for the rTE trials.

### 2.9. Protocol “B”: Torque-Matching Condition

For each rTE and ISO trial, participants were instructed to maintain 40% MVC torque. The movement and stimulation protocols were identical to those of protocol A (Figure 1C).

### 2.10. Data Analysis and Statistics

Mean torque from each protocol contraction was calculated from 500 ms prior to the first stimulation to the end of each contraction [23]. Root mean squared EMG (EMG_RMS_) amplitude was calculated in a 500 ms window that occurred between 6 and 8 s after contraction initiation following the achievement of an isometric steady state. It was ensured that the window selected was matched between corresponding rTE and ISO trials [23]. The EMG_RMS_ of the resting Mmax recorded at the TA and soleus was used to normalize the voluntary TA and soleus EMG, respectively. A paired *t*-test was performed to compare the torque and EMG data between rTE and ISO trials to validate the presence of rTE and activation reduction.

To obtain the reflex parameters, a stimulus-triggered average of the raw TA EMG_RMS_ was generated using Labchart software (Labchart, Pro Modules 2014, version 8). For each protocol, separate rTE and ISO averages were constructed from all stimuli delivered in each condition; therefore, each average was composed of 50 stimuli delivered over 5 contractions (Figure 1D,E). A paired *t*-test was performed to compare reflex characteristics between the rTE and ISO states, including latency, duration, amplitude, and change in average EMG_RMS_ from baseline, in order to characterize changes in the tendon-evoked inhibitory reflex in the rTE and AR state [23]. Baseline was measured as the average EMG_RMS_ in a 300–500 ms window occurring before TStim, and the onset of the stimulation artifact was defined as 0 s. Latency was measured as the time from the initiation of the stimulus artifact to the sharp decrease in baseline EMG_RMS_ occurring at the start of the reflex. Duration was measured as the time occurring from the initial decrease in EMG_RMS_ from baseline at the start of the reflex to when baseline EMG_RMS_ was once again reached. Amplitude was measured as the magnitude of EMG_RMS_ from baseline to the lowest trough in the reflex [23].

Change in average EMG_RMS_ from baseline was measured as the difference between baseline EMG_RMS_ and the average EMG_RMS_ from the duration of the reflex. Paired *t*-tests were also used to detect any differences in the torque produced during MVCs performed before and after the experiment in order to assess any effects of fatigue during the experimental protocol. Descriptive data found in text are reported as means ± standard deviation, while data presented in figures are reported as means ± standard error of the mean. Significance was determined based on α < 0.05.

## 3. Results

### 3.1. Maximum Voluntary Contraction and Voluntary Activation

The mean pretrial MVC torque was 27.6 ± 6.4 Nm, and all participants were capable of achieving near-maximal values for voluntary activation (98.9 ± 1.5%). Following the 20 submaximal contractions, MVC torque was not different from the pretrial values (27.1 ± 6.6 Nm).

### 3.2. Dorsiflexion Torque and Muscle Activity

#### 3.2.1. Activation Matching

**Figure 2:**
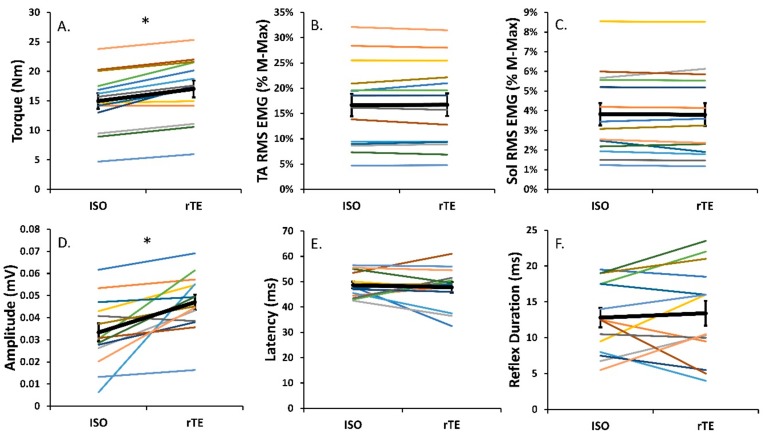
Activation-matching trial. Mean values for each participant across measures (colored lines) and the group mean (black line; error bars indicate standard error of the mean) in the rTE and ISO states. For the activation-matching trial, in the rTE state as compared to the ISO state, there was a 15.0% increase in torque (**A**) and a 22.6% increase in reflex amplitude (**D**) (* *p* < 0.05). There was no significant difference in EMG_RMS_ collected from the tibialis anterior (**B**) or soleus (**C**) reflex duration (**F**) or reflex latency (**E**) between the two states.

Normalized EMG_RMS_ of both TA (*p* = 0.8) and soleus (*p* = 0.6) were not different between rTE and ISO contractions (Figure 2B,C). Following active lengthening, steady-state isometric torque was 15.0 ± 9.7% (*p* < 0.0001) greater than that produced during the purely isometric contractions at the corresponding muscle length and level of activation (Figure 2A). Participants successfully maintained the EMG target level such that iEMG of the TA did not differ in the rTE and ISO contractions (*p* = 0.1; Figure 1B). This indicates indirectly that motor neuron output was similar in both the rTE and ISO states. For the tendon-evoked inhibitory reflex, reflex latency was not significantly different between the rTE and ISO states, with the onset of inhibition occurring at 47.8 ± 7.8 ms following TStim in rTE trials and 48.6 ± 4.8 ms following TStim in ISO trials (*p* = 0.6; Figure 2E). Further, reflex duration (*p* = 0.5; Figure 2F) and reduction in average reflex EMG_RMS_ from baseline were not different when rTE and ISO contractions were compared (*p* = 0.2). However, inhibitory reflex amplitude differed by 22.6 ± 41.8% (*p* < 0.05; Figure 2D) in the rTE state compared to the ISO state.

#### 3.2.2. Torque Matching

**Figure 3:**
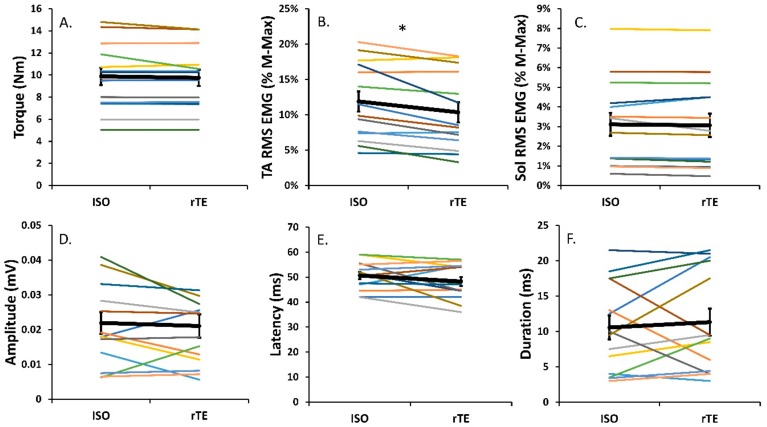
Torque-matching trial. Mean values for each participant across measures (colored lines) and the group mean (black line; error bars indicate standard error of the mean) in rTE and ISO states. For the torque-matching trial, in the rTE state as compared to the ISO state, as expected, there was a 14.4% decrease in TA activation (**B**) (*p < 0.05). There were no differences in torque (**A**), antagonist coactivation (**C**), or any reflex parameter (**D**–**F**) between the two states.

Steady-state isometric torque was not different following active lengthening when the rTE and ISO states were compared (*p* = 0.2 Figure 3A). In the rTE state, however, there was a significant 14.4 ± 13.3% decrease in normalized EMG_RMS_ (*p* < 0.01; Figure 3B) and a 26.9 ± 23.7% decrease in iEMG (*p* < 0.01) for the TA compared to ISO contractions, with no change in antagonist coactivation (Figure 3C). For the tendon-evoked inhibitory reflex, reflex latency was not significantly different between the rTE and ISO states, with the onset of inhibition occurring at 48.0 ± 6.8 ms following TStim in rTE trials and 50.6 ± 5.6 ms following TStim in ISO trials (*p* = 0.1; Figure 3E). Further, reflex duration (*p* = 0.2; Figure 3F), reflex amplitude (*p* = 0.2; Figure 3D), and reduction in average reflex EMG_RMS_ from baseline (*p* = 0.2) were not different when rTE and ISO contractions were compared.

## 4. Discussion

The purpose of this study was to determine how the tendon-mediated inhibitory reflex is modified between the rTE and ISO states through TStim of the tibialis anterior during submaximal activation-matched and torque-matched dorsiflexions. The activation-matched task successfully elicited an ~15% increase in torque in the rTE state compared to ISO, while the torque-matching task resulted in an ~14% decrease in EMG. Our hypothesis of an increased tendon-mediated inhibitory reflex in the rTE state was supported by a 23% increase in inhibitory reflex magnitude in the rTE state compared to the ISO state during the activation-matching task but not the torque-matching task. Therefore, these results support an underlying tension-mediated factor as a plausible explanation for the previously reported decrease in agonist motor neuron pool excitability in the rTE state [15].

In the force-enhanced isomeric steady state, there is greater relative contribution of passive force to total force production, possibly owing to stiffening of the giant molecular spring titin [27]. This increase in passive tension can manifest in rTE contractions in two ways when compared to purely ISO contractions. The first is an activation reduction in torque-matching tasks in which no change in muscle tension occurs [3,29]. The second is an increase in torque, and consequently muscle tension, during activation matching [29,35]. Critical to the present study was the use of both paradigms: activation matching and torque matching. Previous reports of decreases in agonist motor neuron pool [15] and increases in corticospinal [9] excitability have been observed for submaximal and maximal rTE contractions compared to purely ISO contractions. Given the aforementioned investigations [9,15] had similar motor neuron outputs for both ISO and rTE contractions, the alteration in spinal excitability was most likely owing to peripheral sensory inputs. One source of peripheral input upon the motor neuron pool are muscle spindles. While activating their respective Ia afferents via Achilles tendon vibration, no modulatory effect on rTE was observed [35]. Therefore, a tension-mediated factor was speculated as the most likely factor driving the previously reported results [15]. Golgi tendon organs increase Ib afferent firing during periods of increased muscle tension [16] and excite inhibitory interneurons within the spinal cord [17,19]. Excitation of these inhibitory interneurons results in decreased neural output of the agonist motor neuron pool, which can be observed within the surface EMG signal [24]. Through single pulse electrical stimulation of the TA tendon, an inhibitory reflex can be observed in the EMG_RMS_ ~50 ms following stimulation [22,24,25]. Following active lengthening, during an activation-matching task, rTE torque increased by ~15% compared to ISO, which resulted in an ~23% increase in tendon-evoked inhibitory reflex magnitude. The torque-matching task served as a control, i.e., with no changes in torque or muscle tension between rTE and ISO states, no changes in tendon-evoked reflexes should occur. This is particularly important because it ensures any changes observed in the activation-matching trials are indeed due to GTO-mediated reflexes. While there was an ~14% reduction in TA activation (normalized EMG_RMS_), indicating the presence of rTE, negligible differences in muscle tension were detected, and as expected, there was no significant differences in the tendon-evoked inhibitory reflex across the rTE and ISO tasks. The lack of changes in reflex characteristics during the torque-matching task indicates our findings were due to a tension-mediated factor. Therefore, the tension-dependent GTO and the Ib afferent is most likely a key contributor in modulating agonist motor neuron excitability during voluntary control of submaximal contractions in the rTE state.

The results of our investigation are similar to previous reports on rTE and activation reduction. While we found ~15% increase in torque across all 40% activation-matched rTE trials in comparison to ISO, Pinniger and Cresswell [36] found a 12% increase in torque at 25% of maximally activated TA dorsiflexion. The 40% MVC torque-matching task induced an ~14% reduction in activation of the tibialis anterior EMG_RMS_. Other studies reported a similar activation reduction ranging from 5% to 20% [28,29,31]. Additionally, the aim of this study was to elucidate a measurable change in tendon-evoked reflex parameters between ISO and rTE states, where we hypothesized an increase in inhibitory reflex parameters in the rTE state compared to the ISO during the activation-matching protocol but not the torque-matching one. In support of this hypothesis, we found an ~23% increase in inhibitory reflex magnitude in the rTE state compared to the ISO state during the activation-matching task but not the torque-matching one. Under similar premises—decreased torque production in the torque-depressed state eliciting a decrease in tendon-evoked reflex parameters—Sypkes et al. [23] found a 16% reduction in reflex magnitude. The symmetry in the findings of Sypkes et al. [23] and the present study demonstrate a clear relationship between these history-dependent properties of force and the GTO mechanoreceptor. The resulting neural reflex pathway consisting of Ib afferent neurons and spinal inhibitory interneurons provide a plausible explanation to decreased motor neuron pool excitability reported previously [15].

A limitation to the present study was the lack of randomization for the order of protocols A and B. This had the potential to change perceptual threshold and reduce stimulus efficacy throughout the testing session. However, when protocol A ISO was compared to protocol B ISO, there were no differences in reflex parameters (Reflex Latency, Reflex Duration, Reflex Magnitude; Change in TA EMG_RMS_ from Baseline). Thus, the lack of randomization does not appear to influence the results.

## 5. Conclusions

Residual torque enhancement, a history-dependent property of muscle, was present during submaximal activation- and torque-matching tasks. In the isometric steady state following an active lengthening contraction, there was an increase in tendon-evoked inhibitory reflex magnitude compared to purely isometric contractions for the activation-matching task but not the torque-matching task. This observation likely characterizes a tension-dependent increased contribution of tendon-mediated inhibitory feedback on the agonist motor neuron pool and as such may explain—at least partially—the documented decrease in agonist motor neuron excitability during an rTE state [15]. This study provides novel insight into the peripheral contributions of the history dependence of force and bridges the gap between the central nervous system and a property that was once thought to be purely intrinsic to muscle.
